# Identification of Novel PI3Kδ Selective Inhibitors by SVM-Based Multistage Virtual Screening and Molecular Dynamics Simulations

**DOI:** 10.3390/ijms20236000

**Published:** 2019-11-28

**Authors:** Jing-wei Liang, Shan Wang, Ming-yang Wang, Shi-long Li, Wan-qiu Li, Fan-hao Meng

**Affiliations:** School of Pharmacy, China Medical University, Shenyang 110000, China; jwliang@cmu.edu.cn (J.-w.L.); wszyl19950714@163.com (S.W.); wmy940623@163.com (M.-y.W.); 13322489192@163.com (S.-l.L.); lwq363066462@163.com (W.-q.L.)

**Keywords:** virtual screening, PI3Kδ selective inhibitor, SVM, molecular dynamics

## Abstract

Phosphoinositide 3 kinase delta (PI3Kδ) is a lipid kinase that has been implicated in a variety of immune mediated disorders. The research on isoform selectivity was crucial for reducing side effects. In the current study, an optimized hierarchical multistage virtual screening method was utilized for screening the PI3Kδ selective inhibitors. The method sequentially applied a support vector machine (SVM), a protein ligand interaction fingerprint (PLIF) pharmacophore, and a molecular docking approach. The evaluation of the validation set showed a high hit rate and a high enrichment factor of 75.1% and 301.66, respectively. This multistage virtual screening method was then utilized to screen the NCI database. From the final hit list, Compound 10 has great potential as the PI3Kδ inhibitor with micromolar inhibition in the PI3Kδ kinase activity assay. This compound also shows selectivity against PI3Kδ kinase. The method combining SVM, pharmacophore, and docking was capable of screening out the compounds with potential PI3Kδ selective inhibitors. Moreover, structural modification of Compound 10 will contribute to investigating the novel scaffold and designing novel PI3Kδ inhibitors.

## 1. Introduction

Phosphoinositide 3 kinases (PI3Ks) are a family of enzymes involved in cellular functions such as cell growth, proliferation, differentiation, motility, survival, and intracellular trafficking [1,2]. The kinase in the PI3K family is a heterodimer composed of a catalytic subunit p110 and a regulatory subunit p85. According to the structural characteristics of PI3Ks and substrate molecules, the PI3K family can be divided into three types, namely, type I, type II, and type III, where the different types of PI3Ks perform different functions. Type I PI3Ks are mainly expressed in immune cells and hematopoietic cells. They participate in the signal transduction of the BCR in B cells, and control the development and maturation of B cells in the body. Phosphoinositide 3 kinase delta (PI3Kδ) belongs to type I PI3Ks and is a lipid kinase that has been implicated to play a key role in a variety of immune mediated disorders such as asthma, rheumatoid arthritis, and other inflammatory diseases [3]. The inhibitors that selectively target PI3Kδ show lower toxicity than pan PI3K inhibitors due to the fewer off target effects in nonhematopoietic tissues [4]. In 2014, idelalisib (Compound 1), a PI3Kδ selective small molecule inhibitor, received the FDA (Food and Drug Administration)’s approval as a new treatment of chronic lymphocytic leukemia (CLL).

**Figure 1:**
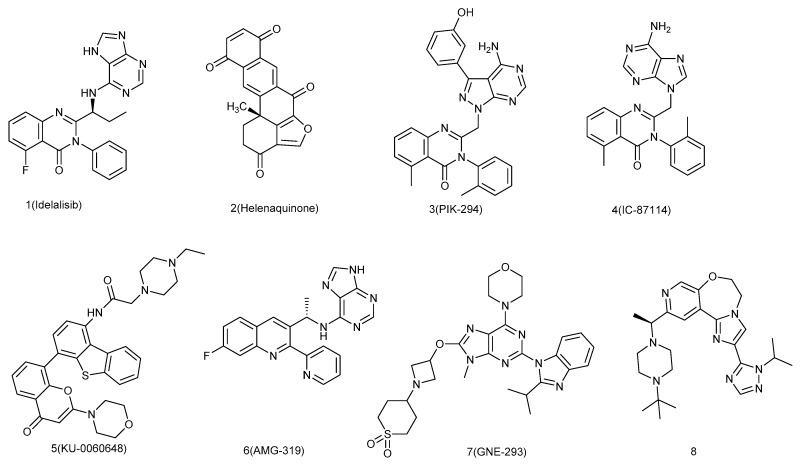
Chemical structure of the phosphoinositide 3 kinase delta (PI3Kδ) inhibitors.

However, the reported selective inhibitors of PI3Kδ are weak with respect to structural diversity, and there are few methods to find them (Figure 1). The difference in the structural characteristics between selective and nonselective PI3Kδ inhibitors has not been systematically summarized, and the active sites between the PI3K family’s subtypes exhibit a high similarity. They possess a similar skeleton, that is, a quinazoline or quinoline linked with an indazole by short hydrocarbon chains. Studies on the selectivity of PI3Kδ inhibitors have suggested that the indazole of the inhibitors occupied a selectivity pocket formed by Trp760 and Trp780 of PI3Kδ, which is called the “tryptophan shelf”. However, a large number of studies have shown that indazole can inhibit cell proliferation and promote apoptosis, which is an important cause of cytotoxicity and nervous system damage. [5,6,7]. Although some researchers transformed the selective PI3Kα inhibitors into selective PI3Kδ inhibitors so that the “tryptophan shelf” is destroyed by arg770 of PI3Kα [8], they did not consider the selectivity of other PI3K subtypes (such as β, γ, etc.). Therefore, it is of great importance to search for a new skeleton with the selective inhibition of PI3Kδ. 

**Figure 2:**
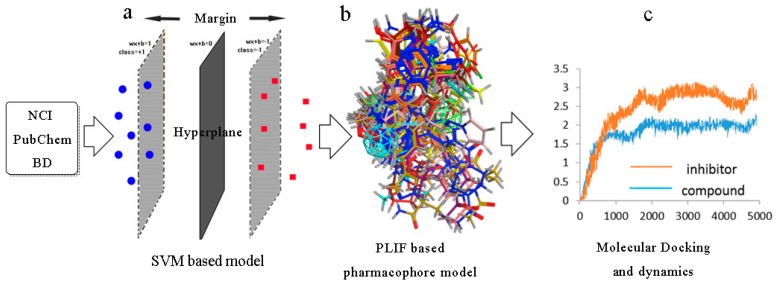
The multistage virtual screening method, (**a**) support vector machine SVM-based model; (**b**) PLIF-based pharmacophore model; (**c**) molecular docking and dynamics. BD, the BindingDB.

In order to discover PI3Kδ selective inhibitors with a novel skeleton to expand the structural diversity of selective PI3Kδ inhibitors, a virtual screening method based on the support vector machine (SVM) was carried out in this work. By decomposing the PI3Kδ selective inhibitors into descriptor information, the compounds with favorable ADME (It refers to the absorption, distribution, metabolism and excretion of foreign chemicals) properties could be screened out. However, each virtual screening method has drawbacks when used alone. The difficulties of reducing the false positive (FP) rate and increasing the isoform selectivity were solved by combining protein–ligand interaction fingerprint (PLIF) pharmacophore and docking methods. In the current research, we report the SVM based multistage virtual screening of compounds from the NCI database, PubChem, and the BindingDB, and the biological evaluation of the hit compound (Figure 2).

## 2. Results and Discussion

### 2.1. Establishment and Evaluation of the SVM Model

The Calculate Descriptors function in the MOE2016 software (MOE2016.10, Chemical Computing Group Inc, Montreal, QC, Canada) includes geometrical, topological, and electronic properties. The constructed PI3Kδ inhibitor and noninhibitor training set contained 477 inhibitors and 8612 decoys, respectively. The 435 molecular descriptors of the training set compounds were calculated by the Calculate Descriptors function. After filtering the descriptors with the GA SVM method, 34 of the 435 molecular descriptors were selected for the further evaluation and validation using the SVM model, and these molecular descriptors were divided into eight groups, as shown in Table 1. The Grid Search result showed that when the values of the parameter C and g were 8 and 0.01, respectively, the LibSVM model demonstrated the best accuracy.

Ten fold cross validation was used to evaluate the constructed SVM model by using the training set. The evaluation results are shown in Table 2. Among the 477 PI3Kδ inhibitors, 466 compounds (true positive (TP)) were correctly predicted, whereas 11 compounds (false negative (FN)) were wrongly predicted. The sensitivity (SE) was 97.7%. As for the 8612 PI3Kδ noninhibitors, 8509 compounds were correctly (true negative (TN)) predicted and 103 compounds (false positive (FP)) were incorrectly predicted. The specificity (SP) value was 98.8%. The overall accuracy (Q) was 98.7%, which demonstrated that the constructed SVM model was appropriate for the inhibitor and noninhibitor compounds in the training dataset. Then the independent test set was used to validate the predictive ability. The prediction and evaluation results are shown in Table 2. Among the 50 PI3Kδ inhibitors in the test set, 42 compounds (TP) were correctly predicted with SE values of 84%. As for the 50 PI3Kδ noninhibitors, 47 compounds (TN) were correctly predicted with SP values of 94%. Of all the 100 compounds in the test set, 89 compounds were correctly predicted, with Q values of 89%. Validation and evaluation results of the ten-fold cross validation and independent test indicated that the constructed SVM model fitted with both training set and test set and possessed high accuracy and predictive ability.

### 2.2. Validation and Evaluation of the PLIF Pharmacophore Model

**Figure 3:**
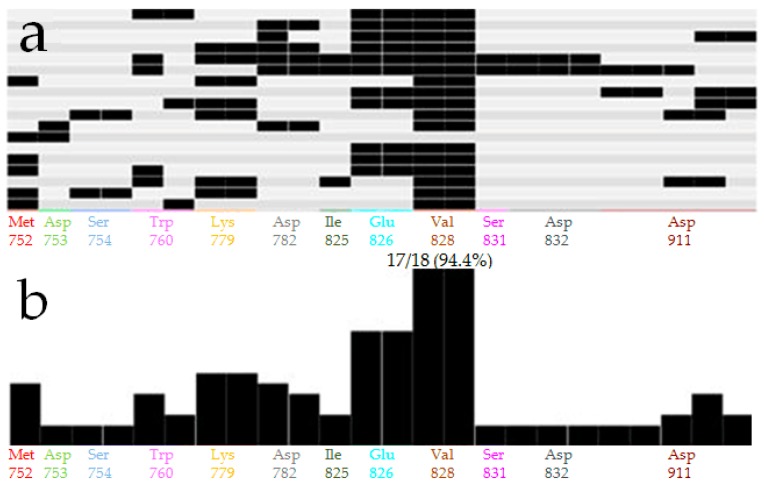
The PLIF pharmacophore model. (**a**) Barcodes of the amino acid interaction fingerprint generated by the MOE2016 software. (**b**) Population mode of the barcode fingerprint.

The PLIF technology in MOE2016 was carried out for building the pharmacophore model of the PI3Kδ inhibitors. The PLIF tool is a method for summarizing the interactions between ligands and proteins using a fingerprint scheme [9,10]. As depicted in Figure 3, 94.4% of inhibitors formed an interaction with the residue Val828, which indicated that the residue Val828 was an important site for PI3Kδ inhibitor binding. Moreover, the inhibitors also formed a strong interaction with the residues Glu826, Lys729, and Met752. Based on these interaction fingerprints, three pharmacophore cores were elicited. 

The performances of the three models were further evaluated by a training set to select the best one. The results of the evaluation are shown in Table 3, where the pharmacophore model 2 (detailed in Table S1) with the highest yield value of 89.7% showed that 428 of the 477 PI3Kδ inhibitors were correctly predicted (yield represents the percentage of predicted compounds in known inhibitors). In addition, 1755 of 8612 PI3Kδ non-inhibitors were also matched properly, which produced the low hit rate (percentage of known inhibitors in predicted compounds). All the pharmacophore models could differentiate the PI3Kδ inhibitors from noninhibitors to some degree. However, these models generated a large number of false positive prediction results, this being the reason that the multistage virtual screening method was adopted.

### 2.3. Determining Docking Parameters and Validation

The crystal structure of the PI3Kδ complexed (PDB ID: 2wxf) with 2-((9h-purin-6-ylthio) methyl)-5-chloro-3-(2-methoxyphenyl) quinazoline-4(3h)-one was chosen as the reference structure of the receptor since it has the highest resolution among all the PI3Kδ crystal structures (1.9 Å). Twenty PI3Kδ inhibitors with IC_50_ values were docked to PI3Kδ using the GoldScore, ChemScore, ASP, and PLP values, respectively. The order of ChemScore values demonstrated the highest consistency of the experimental IC_50_. Finally, 391 of 477 PI3Kδ inhibitors were correctly predicted with a yield of 82.0%; the docking process can distinguish PI3Kδ inhibitors from the training set. As was the case with the pharmacophore based virtual screening, 932 of 8612 decoys were wrongly predicted as the PI3Kδ inhibitors. Therefore, selecting the multistage virtual screening method makes sense because of the high TP (932) experienced as a result of the docking virtual screening.

### 2.4. Validation and Evaluation of the Performance of the Multistage Virtual Screening Method

During the process of validating and evaluating the multistage virtual screening method, the SVM, pharmacophore, and docking virtual screening methods were individually performed for screening the PI3Kδ inhibitors from the validation set. To evaluate the prediction accuracy of the multistage virtual screening method, the yield, hit rate, and enrichment factor (ratio of hit rate to the percentage of known inhibitors in the validation set) were assessed and are presented in Table 4. Finally, the SVM method was selected as the first filter, while the pharmacophore and docking methods were selected as the second and third ones, respectively.

Then, the SVM method was combined with the pharmacophore method to investigate the speed and accuracy of the screening validation set. After the SVM virtual screening method, 133 of 175 PI3Kδ inhibitors were correctly predicted and 131,098 of 132,914 decoys were correctly predicted, so the set composed of 1816 decoys and 133 PI3Kδ inhibitors was selected as the initial set for the combined virtual screening method. For the SVM–pharmacophore virtual screening method, 107 of 133 PI3Kδ inhibitors were correctly predicted with a yield of 80.4%, the values of hit rate and enrichment factors were 27.7% and 118.32, respectively, and the time cost in this combined method was 0.93 h. The combination of the two methods was better than both the SVM method and the pharmacophore method alone in terms of speed and accuracy (higher hit rate and enrichment factor). This was because the false positive of the SVM method (FP = 1816) in the screening process was far less than that of the pharmacophore method (FP = 21,322) and method 3 (FP = 15,266). The introduction of the SVM method as the first level filter increases the proportion of correct positive results in all predicted positive results, therefore the values of hit rate and enrichment factor increase greatly to facilitate distinguishing the positive and negative element.

Finally, the multistage virtual screening method based on the SVM, pharmacophore, and docking models was performed to screen the PI3Kδ inhibitors in the validation set. The combined method obtained a set of 651 PI3Kδ noninhibitors and 128 inhibitors; after performing the docking virtual screening method, the 112 PI3Kδ inhibitors and 601 noninhibitors were correctly predicted. The yield, hit rate, and enrichment factors were 70.9%, 75.1%, and 301.66, respectively, and the whole multistage virtual screening process took 1.72 h. The multistage virtual screening method obtained the highest hit rate as compared to the other methods which was up to 80.06%. With the addition of the docking method, the accuracy of the virtual screening was greatly improved over the combined method, although it came with a slight loss of the yield (70.9%). Moreover, because the fastest method, SVM, initially ignored too many negative results, the screening time of the slower pharmacophore and docking methods was significantly reduced. We found that all of the separate approach virtual screening methods were not as efficient and accurate as the multistage method for the following aspects. (i) All of the separate approach virtual screening methods experienced the problem that positive results accounted for a high proportion of predicted positive results, which resulted in a low hit rate and enrichment factor (particularly in the pharmacophore and docking methods, where the hit rate and enrichment factor were less than 2%) and which were untrustworthy and meaningless in the virtual screening results. (ii) In addition to the problem of being a time consuming process, the ligand based and structure based method made it also difficult to find compounds with novel scaffolds when screening the large chemical library containing millions or even tens of millions of compounds by performing the pharmacophore and docking methods. Furthermore, using the SVM method alone resulted in overfitting between the constructed SVM model and the training set, which could not be verified and avoided by subsequent methods.

### 2.5. Virtual Screening Using the Multistage Virtual Screening Method for Obtaining the PI3Kδ Inhibitors from the NCI Database

**Scheme 1:**
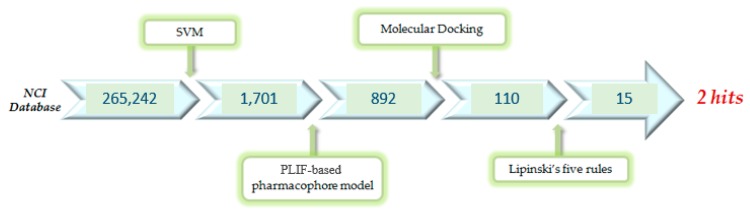
The results of the multistage virtual screening.

**Figure 4:**
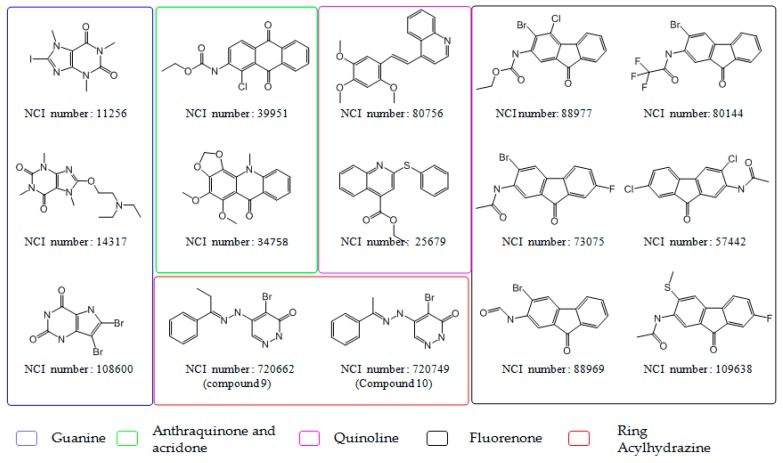
The 15 compounds obtained by the multistage virtual screening.

The multistage virtual screening method (SVM-pharmacophore-docking) was utilized to screen the PI3Kδ inhibitors from the NCI database (containing 265,242 compounds). From these, 1701 positive compounds were obtained from the first SVM virtual screening approach. These compounds continued to be filtered using the pharmacophore method, and 892 positive compounds were screened out. After implementing the GOLD docking approach, we ended up with 110 compounds (Scheme 1). Finally, we screened the compounds using the drug like rule (i.e., Lipinski’s five rules), 15 compounds were screened out (detailed in Table S2), and the compounds were divided into five groups according to their skeletons as shown in Figure 4. The compounds in the blue box all possessed guanine, which was the main skeleton of the PI3Kδ selective inhibitor IC-87114. The compounds in the green, pink, and black boxes also possessed similar skeletons with the reported PI3Kδ inhibitors such as helenaquinone, LY294002, seletalisib, resveratrol, and KU-0060648. Finally, the two compounds in the red box possessed a novel skeleton. It indicated the rationality of the screening results that most hit compounds possessed a similar skeleton with the reported PI3Kδ inhibitors.

### 2.6. The PI3Kδ Inhibition Activity of the 15 Hit Compounds

The chemiluminescence method was used to determine the inhibitory effect of the 15 hit compounds on PI3Kδ (Table 5). Most of the hit compounds showed inhibitory activity against PI3Kδ with a hit rate of 73.3%. The compound 14317, Compound 9, and Compound 10 exhibited better activity against PI3Kδ. For 14317, the reason for its high activity was probably the long side chain on its guanine, which was the same as the reported PI3Kδ selective inhibitor Compound 1 (idelalisib) and Compound 7 (GNE-293). For Compounds 9 and 10, the reason the pyridazinone skeleton made them so active requires further study. Finally, the two compounds with the novel skeleton were selected for the subsequent molecular dynamics (MD) simulations.

### 2.7. Validation and Comparison of the Binding Stability between the Screened Compounds and the PI3Kδ Receptor Protein

**Figure 5:**
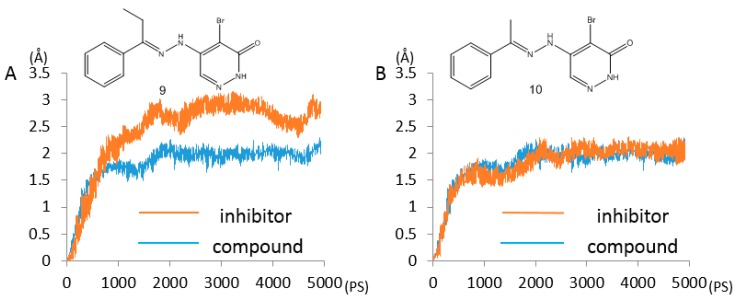
The molecular dynamics results of the inhibitor and the two screened compounds, (**A**) Compound 9 and (**B**) Compound 10. The RMSD of the inhibitor–CDK2 complex is painted in brown, and the two screened compounds are painted in blue.

The molecular dynamics results of the two hit compounds are shown in Figure 5. The RMSD plot of the inhibitor–PI3Kδ complex exhibits a sharp upward trend in the first 1.5 ns; afterward, the value of the RMSD plot is flat around 2 Å until the end of the simulation. Of the two compound–PI3Kδ complexes, only Compound 10 shows the same stability to the PI3Kδ receptor as an inhibitor. In Figure 5B, the RMSD plot of Compound 10–PI3Kδ reaches 2 Å in 1.4 ns and it keeps fluctuating a little around that value until the end of 5 ns. Another compound demonstrated higher RMSD values than the inhibitor–PI3Kδ complex, so it is unstable when it binds to the ATP competitive pocket of the PI3Kδ receptor. 

**Figure 6:**
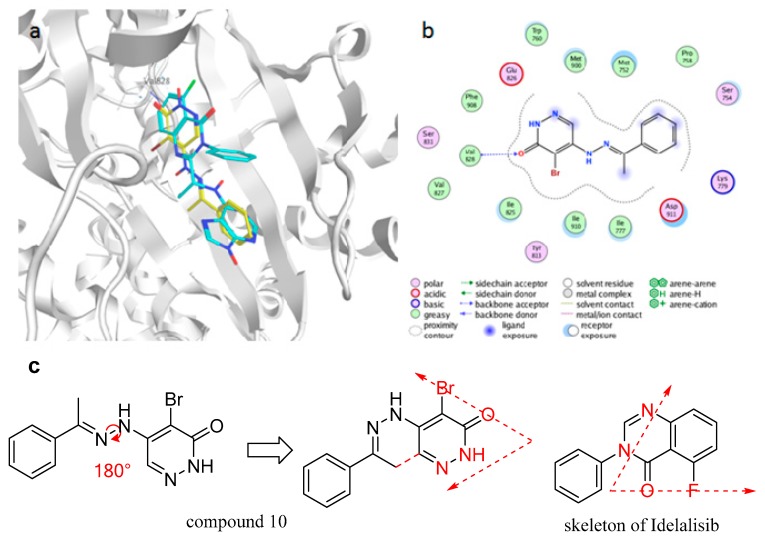
(**a**) the superposition between Compound 10 (yellow) and idelalisib (Compound 1, blue) in the PI3Kδ active pockets; (**b**) ligand interaction (planar projection of (**a**)); (**c**) the comparison between Compound 10 and the Skeleton of idelalisib.

The binding mode of Compound 10 with PI3Kδ is shown in Figure 6. Compound 10 formed the hydrogen bonds with Val 828, which was indicated as an important site for PI3Kδ inhibitor binding. In the active pocket of the PI3Kδ, the pyridazinone of Compound 10 overlapped with the quinazolinone of idelalisib, and the aromatic ring linked by hydrazone overlapped with the adenine. Since the single bond of the hydrazone is rotatable, Compound 10 can also be drawn as the structure in Figure 6c. Coincidently, this structure was very similar to the quinazolinone skeleton of idelalisib, especially when the hydrazone and the pyridazinone were buckled. Moreover, the order and the relative position of the heteroatoms in these two structures were almost the same (dotted arrow in Figure 6c). This not only confirmed the rationality of the screening result but also provided a new idea for structural optimization. 

### 2.8. The PI3Kδ Inhibition Activity of Compounds 9 and 10

**Figure 7:**
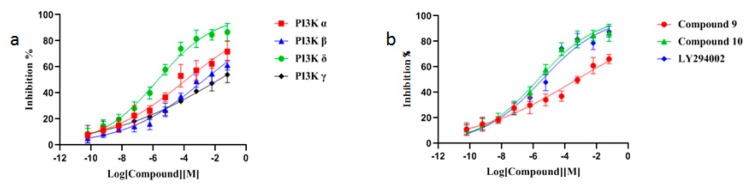
(**a**) The PI3Ks inhibition activity of Compound 10. (**b**) The PI3Kδ selective inhibition of Compound 9, Compound 10, and LY294002.

The chemiluminescence method was used to determine the inhibitory effect of Compounds 9 and 10 on PI3Ks kinase, and LY294002 was used as a positive control. The inhibitory effect of Compound 10 on PI3Kδ was comparable to that of LY294002 with the IC_50_ value of 18.93 μM (Table 6, Figure 7b). Moreover, in the PI3K isoform selectivity experiment, Compound 10 showed significant selectivity (Table 6, Figure 7a). The above results indicate that the multilevel screening model worked as expected. As a ligand based screening method, SVM selected small molecules with PI3Kδ inhibitory activity. These small molecules contain nonselective inhibitors and compounds with poor ADME properties (e.g., high intramolecular energy). The subsequent PLIF-pharmacophore model, considered the interaction of PI3Kδ kinase with selective PI3Kδ inhibitors, selected small molecules with selective PI3Kδ inhibitory activity. Finally, molecular docking and molecular dynamics simulations screened the molecules with the smallest intramolecular energy.

## 3. Materials and Methods

### 3.1. Compound Collection and Dataset Construction

A total of 702 PI3Kδ inhibitors (IC_50_ ≤ 10 μM) were collected from the BindingDB and the Directory of Useful Decoys databases’ positives (207 from the BindingDB, 495 from DUD). In addition, 100 PI3Kδ noninhibitors were collected from the BindingDB with the IC_50_ ≥ 100 μM (the structures of 702 PI3Kδ inhibitors and 100 PI3Kδ noninhibitors are shown in Table S3). The two cutoff values (IC_50_ ≤ 10 μM and IC_50_ ≥ 100 μM) minimize the risk of including potential PI3Kδ inhibitors in the negative group and reduces the number of false positives during virtual screening. The decoys were selected so that they would have similar physical properties with PI3Kδ inhibitors but be chemically distinct from them [11,12]. In detail, a parallel strategy to Shoichet’s and Garcia Vallve’s was applied to develop the decoy sets from the NCI database, PubChem, and the BindingDB. First, Tanimoto coefficients between a set of 651 known PI3Kδ inhibitors from the BindingDB (positives from the training set and positives from the validation set) and 265,242 compounds from the NCI database, 192,845,102 compounds from PubChem, and 442,444 compounds from the BindingDB were calculated based on an extended connectivity fingerprint (ECFP) similarity analysis. Compounds with Tanimoto coefficients of less than 0.5 with any of the selected PI3Kδ inhibitors were selected. Second, five physical properties including molecular weight, number of hydrogen bond donors and hydrogen bond acceptors, number of rotatable bonds, and logP were defined by using DecoyFinder 2.0 (Grup de Recerca en Nutrigenòmica, Departament de Bioquímica i Biotecnologia, Universitat Rovira i Virgili, Campus de Sescelades, C/Marcel.lí Domingo s/n, 43007, Tarragona, Catalonia, Spain) for the PI3Kδ inhibitors collected from the previous step. Then, the compounds with five calculated physical properties similar to any of the PI3Kδ inhibitors and with a Tanimoto coefficient higher than 0.5 were further selected in a decoy dataset. Then, 9982 decoys from NCI, 30,259 decoys from PubChem, and 102,655 decoys from the BindingDB were used to construct a training set and a validation set. Finally, the datasets included the training set (477 positive compounds together with the 8612 decoys obtained from NCI), the test set (50 positive compounds together with the 50 negative compounds (non inhibitor) from the BindingDB), and the validation set (175 positive compounds together with 9982 decoys from the BindingDB and 30,259 decoys from PubChem).

### 3.2. SVM Modeling

First, the 435 descriptors for the compound sets were calculated using the MOE2016 software after energy minimization [13]. Then, the 435 descriptors were pre processed by the data filter plugin in Python to remove redundant data: (i) descriptors with large quantities of null, constant, and zero values were eliminated; (ii) descriptors with very small standard deviation values (stdev < 0.5%) were eliminated; and (iii) descriptors with a high correlation to others (correlation coefficient > 0.95) were eliminated. The remaining molecular descriptors were normalized to a range of 0 to +1, which was necessary since the different ranges of descriptor values influence the quality of the SVM model generated. The GA-SVM method was utilized to perform a further feature selection process to the normalized descriptors. Finally, the LibSVM software developed by Chang C. and Lin C. [14] was used to establish the SVM classification model based on descriptors selected from GA-SVM.

### 3.3. Pharmacophore Modeling

The 18 PI3Kδ proteins with the inhibitor were collected from the PDB database, and all the superposed ligand-receptor complexes were aligned in the MOE software for calculating the PLIF rows. These PLIF rows were then utilized for generating the amino acid interaction fingerprints. The interaction fingerprints were then generated by the PLIF Setup function in the Database Viewer panel, all the interactions having the min score 1 and score 2 of 0.5 kcal/mol and 1.5 kcal/mol, respectively. Then, the Query Generator tool was carried out for generating pharmacophore queries based on the frequencies of the protein-ligand contacts. The max Radius was set to 3, the Feature Coverage was set to 25–75%, Excluded Volumes was set to ON, and the other parameters were kept at their default values.

### 3.4. Molecular Docking Study

The optimization of the docking parameters and the selection of the function score have a great impact on the final results, and they were considered important in the process of docking-based virtual screening. In order to determine the best docking parameters, a self-docking of the five active compounds into PI3Kδ’s active site was performed. The Efficiency was set to Virtual Screening (30%); the Early Termination was set to OFF; the Placement and Refinement were set to 30 and 10, respectively; the Allow rotatable waters and Internal ligand H-bonds were turned to OFF; the behavior of protonated carboxylic acids during docking was turned to rotated; the Flip Ring corner, Planar N, and Pyramidal N were turned on; GA Parameters were set to auto; and the other parameters used their default values.

The ChemScore module in the MOE2016 software was used to perform the molecular docking study and analyze the interaction between the ligand and receptor. The targeted protein was corrected, protonated, tethered, and stage minimized by the QuickPrep function in the MOE panel; the Structure, Protonate3D, and ASN/GLN/HIS Flips were set to on, the waters farther than 4.5 Å from ligand or receptor were deleted. The binding pocket was defined near the ATP competitive site within 6.0 Å, making sure that the space was large enough for accommodating the ligand scale. When selecting function scores, 20 inhibitors with IC_50_ values were docked into the binding pocket by using GoldScore, ChemScore, ASP, and PLP function score; the discounted cumulative gain (DCG) algorithm was used to examine the consistency between the experimental IC_50_ and different score functions [15]. The reli refers to the pIC_50_ and the IDCG refers to the ordered IC_50_ values. The closer the normalized discounted cumulative gain (NDCG) value is to 1, the better the consistency between the IC_50_ and function score. Finally, the selected function score was used to evaluate the affinity between the ligand and the compound as follows:

(1) $$NDCG_{P}=\frac{DCG_{P}}{IDCG_{P}},$$

(2) $$DCG_{P}=rel_{1}+\sum_{i=2}^{p}\frac{rel_{i}}{{log}_{2}i}.$$

### 3.5. Molecular Dynamics

Preliminary MD simulations for the model protein were performed using the program NAMD (v2.9, Theoretical Biophysics Group University of Illinois and Beckman Institute 405 N. Mathews Urbana, IL, USA), and all files were generated using visual molecular dynamics [16]. NAMD is a freely available software designed for the high performance simulation of large biomolecular systems. During MD simulation, the minimization and equilibration of the original and docked protein was in a 15 Å^3^ size water box, and an Amber 10 EHT force field file was applied for energy minimization and equilibration together with a Gasteiger–Hückel charge using the Boltzmann initial velocity. The integrator parameters included 2fs/step for all rigid bonds and non-bonded frequencies were selected for 1 Å, and full electrostatic evaluations were conducted for 2 Å and used with ten steps for each cycle [17]. The pressure was maintained at 101.325 kPa using the Langevin piston and temperature was controlled to 310 K using Langevin dynamics. The covalent interactions that exist between hydrogen and heavy atoms were observed to be constrained as revealed by the SHAKE/RATTLE algorithm [18]. Finally, a 5 ns MD simulation for docked protein was utilized to compare and verify the binding affinity and stability of the ligand receptor complex.

### 3.6. Cell Free Detection of PI3Kδ Kinase Activity

The inhibitory activities of inhibitors against PI3Kδ were determined by the ADP-Glo assay. The recombinant PI3Kδ kinase was diluted to 2.2 μg/mL using reaction buffer (50 mM HEPES, pH 7.5, 10 mM MgCl_2_, 0.1 mg/mL BSA, 2 mM DTT, 1% DMSO), and ATP was diluted with reaction buffer (10 mM) to 250 μM, and the test compound and positive drug (idelalisib) were formulated into four concentration-gradient solutions (6 × 10^−2^ M, 6 × 10^−4^ M, 6 × 10^−6^ M, 6 × 10^−8^ M). The reaction was started by sequentially adding 2 μL of an ATP solution, 1 μL of a drug solution, and 2 μL of an enzyme solution in 96 wells. The assay was conducted for 1 h at 37 °C before the addition of 5 μL ADP-Glo reagent and incubation for 40 min at room temperature. A 10 μL amount of kinase detection reagent (Promega, Beijing, China) was added and incubated for 30 min at room temperature before the luminescence value was measured using a chemiluminescence module of a full-wavelength multi-function microplate reader (PerkinElmer, Singapore).

## 4. Conclusions

In this investigation, a multistage virtual screening method including SVM, pharmacophore, and docking methods was utilized for screening PI3Kδ inhibitors from the NCI dataset. Initially, the SVM, pharmacophore, and docking methods were evaluated and optimized individually with the training and test sets constructed using the BindingDB, PubChem, and NCI databases. In all, 15 compounds were screened out, 11 of which showed inhibitory activity against PI3Kδ. Compounds 9 and 10 with a new skeleton showed good activity with IC_50_ values of 72.18 μM and 18.93 μM, respectively. Finally, Compound 10 was shown stable in the ATP-binding site and exhibited high isoform selectivity. Our study proved that the multistage virtual screening method combining SVM, pharmacophore, and docking methods was able to screen out the compounds with potential PI3Kδ selective inhibitors. Moreover, structural modification of Compound 10 will be carried out for investigating the mechanism and structure-function relationship of the novel scaffold and designing novel PI3Kδ inhibitors.

## Figures and Tables

**Table 1:** The 34 molecular descriptors filtered by the GA SVM method for building the SVM.

Descriptor Class	Descriptors	Number
Physical Properties	radius, density, diameter	3
Subdivided Surface Areas	SMR_VSA1,SMR_VSA2,SMR_VSA3, SlogP_VSA3,SlogP_VSA5,SlogP_VSA7,SlogP_VSA9,SlogP	8
Atom Counts and Bond Counts	a_IC, lip_don, b_rotN, b_double	4
Kier and Hall Connectivity and Kappa Shape Indices	chi1, KierA2, KierA3	3
Adjacency and Distance Matrix Descriptors	BCUT_SLOGP_1, BCUT_SMR_1, GCUT_SMR_0, VDistMa	4
Pharmacophore Feature Descriptors	vsa_don	1
Partial Charge Descriptors	PEOE_VSA-2, PEOE_VSA_PPOS, PEOE_VSA_NEG, Q_PC+, PEOE_VSA_FPOS, Q_VSA_PPOS, Q_VSA_FPPOS, Q_RPC-,PC+	9
Surface Area, Volume, and Shape Descriptors	opr_violation, zagreb	2

**Table 2:** The evaluation and validation results of the ten fold cross validation and independent test.

Method	Positive			Negative			
Tenfold cross-validation Independent test	TP	FN	SE (%)	TN	FP	SP	Q (%)
	466	11	97.7	8509	103	98.8	98.7
	42	8	84	47	3	94	89

TP, true positive; FN, false negative; SE, sensitivity; TN, true negative; FP, false positive; SP, specificity; Q, overall accuracy.

**Table 3:** The evaluation and validation results of the three pharmacophore models generated by PLIF.

Pharmacophore Models	TP	FP	Yield (%)	Hit Rate (%)
1	389	2411	81.5	9.74
2	428	1755	89.7	11.6
3	371	2127	77.7	8.21

**Table 4:** Validation and evaluation of the various virtual screening methods using the validation set that contained 175 known PI3Kδ inhibitors and 132,914 decoys.

Method	Predicted Positive	Hits	Hit Rate (%)	Enrichment Factor	Yield (%)	Time (h)
SVM	1949	142	11.1	48.50	81.1	0.25
Pharmacophore	23,265	107	1.02	4.68	88.0	7
Docking	17,501	137	1.61	6.87	73.7	362.23
SVM-Pharmacophore	1949/779	142/298	27.7	118.32	80.4	0.93
SVM-Pharmacophore-Docking	1949/779/346	142/298/277	75.1	301.66	70.9	1.72

**Table 5:** The PI3Kδ inhibition activity of the 15 compounds.

NCI Number	PI3Kδ IC_50_ (μM)	Molecular Docking Score
11256	−	−3.0550
14317	28.14	2.6691
108600	−	−4.4181
39951	522.47	−3.2204
34758	481.02	1.1669
80756	192.24	−2.0794
25679	289.01	−2.4986
88977	−	3.3571
80144	809.21	4.3935
73075	247.58	−5.6527
57442	500.29	−4.9110
88969	−	−2.2508
109638	169.18	−3.4637
720662 (Compound 9)	72.18	2.8963
720749 (Compound 10)	18.93	−4.8226

**Table 6:** The PI3Ks inhibition activity of LY294002 and Compound 10.

Compound	PI3K IC_50_ (μM)			
	α	β	δ	γ
LY294002	0.74	1.53	3.49	3.51
Compound 10	75.01	187.60	18.93	226.3

## Supplementary Materials

The following are available online at https://www.mdpi.com/1422-0067/20/23/6000/s1.
